# Identification of targets of clinical FOXA1 mutants involved in EMT and immune suppression in prostate cancer

**DOI:** 10.1038/s42003-026-10523-2

**Published:** 2026-06-23

**Authors:** Yuxin Dong, Mingyu Liu, Ruogyu Gao, Muqing Li, Junyang Zhai, Changmeng Cai, Shuai Gao

**Affiliations:** 1 Department of Cell and Molecular Physiology, New York Medical College, Valhalla, NY USA; 2 Center for Personalized Cancer Therapy, University of Massachusetts Boston, Boston, MA USA; 3 Department of Biology, University of Massachusetts Boston, Boston, MA USA; 4 Department of Urology, Brigham and Women’s Hospital and Harvard Medical School, Boston, MA USA

**Keywords:** Prostate cancer, Gene expression

## Abstract

FOXA1 is a critical transcription factor in prostate cancer (PCa) progression, functioning as a pioneer factor for the androgen receptor (AR). Its expression is elevated in advanced stages of PCa and plays a central role in oncogenic reprogramming. Recurrent coding mutations in the forkhead DNA-binding domain (FKHD) of FOXA1 become increasingly prevalent in drug-resistant metastatic PCa, particularly clustering in the Wing2 region of FKHD. While recent studies demonstrated that these mutations promote PCa progression, the downstream targets of FKHD mutants remain poorly understood. In this study, we employed three analytical approaches to elucidate these targets: (i) traditional transcriptomic analyses, (ii) chromatin structure–based super-enhancer (SE) profiling, and (iii) immune deconvolution. We identified HPSE, an enzyme that degrades heparan sulfate, as a druggable target activated by FKHD mutants both in vitro and in vivo. SE profiling further revealed that FKHD mutants can reprogram the SE landscape, with FOXP1 emerging as a consistently SE-driven and upregulated gene across multiple FOXA1 mutants. Additionally, FOXA1 mutations were associated with immune suppression in PCa patient samples, and *B2M*, a key gene in antigen processing and presentation, was notably downregulated in FKHD-mutant tumors. Together, our findings provide distinct insights into how FOXA1 FKHD mutations drive PCa progression and highlight potential therapeutic targets in tumors harboring these mutations.

## Abstract (web summary)

FOXA1 FKHD mutations drive prostate cancer by activating HPSE, reprogramming super-enhancers to upregulate FOXP1, and suppressing immune pathways including B2M, revealing distinct mechanisms and therapeutic vulnerabilities in mutant tumors.

## Introduction

FOXA1 (Forkhead box A1) is a key pioneer transcription factor that plays a critical role in chromatin remodeling and transcriptional regulation in prostate epithelial cells^1^. Recurrent coding mutations in FOXA1 are detected in approximately 4% of primary prostate cancers (PCa), with a marked increase in frequency (10–13%) in metastatic castration-resistant prostate cancer (mCRPC)^2–5^. The majority of these mutations localize to the Wing 2 region (H247-E269) within the C-terminal portion of the Forkhead DNA-binding domain (FKHD), a known hotspot for both missense and insertion/deletion (indel) alterations, accounting for over 50% of all FOXA1 coding mutations.

FKHD missense mutations were shown to reduce FOXA1 binding at classical androgen receptor (AR)-regulated enhancers, leading to diminished AR transcriptional activity. However, these same mutations promote activation of the epithelial–mesenchymal transition (EMT) program through preferential binding to non-canonical FKHD motifs^6,7^. Recent identification of Semaphorin 3 C (SEMA3C), a pro-proliferative and EMT-associated oncogene, as a direct target of mutant FOXA1 further supports the role of these mutations in driving PCa aggressiveness^8^. Additional studies have shown that FKHD mutations increase FOXA1 nuclear mobility and drive both cell proliferation and organoid growth, suggesting a multifaceted role in tumor progression^3^. A recent comprehensive genomic analysis of 5,014 PCa cases stratified FOXA1 alterations into four major classes and seven subclasses, underscoring the importance of molecularly informed stratification for precision treatment approaches^2^. Most recently, a study using transgenic mouse models demonstrated that distinct classes of FOXA1 mutations confer different transcriptional outputs and drive PCa development through divergent biological mechanisms^9^.

Despite its oncogenic importance, FOXA1 lacks a druggable domain, making it challenging to target directly. This has prompted efforts to identify FOXA1-dependent downstream effectors that may represent actionable vulnerabilities. Previously, we reported that FKHD missense mutants activate MET, rendering mutant cells and xenografts sensitive to the FDA-approved MET inhibitor crizotinib^7^. Additionally, the WNT inhibitor XAV939 has been shown to suppress the invasive phenotype associated with FOXA1 truncating mutations^3^.

Notably, FKHD mutations in PCa patients are associated with significantly higher FOXA1 transcript levels, comparable to those observed in cases with FOXA1 amplification^2^. However, whether these mutations drive distinct transcriptional programs remains largely unknown. In the present study, we compared FKHD mutant overexpression to wild-type (WT) FOXA1 overexpression to delineate the transcriptional networks directed by FKHD mutants. Transcriptomic profiling of xenografts harboring FKHD mutations identified HPSE as a putative therapeutic target capable of attenuating the aggressive phenotype of mutant cells. We also demonstrate that FKHD mutations reprogram super-enhancers (SEs) and activate expression of key oncogenes implicated in PCa progression. Finally, we provide evidence that FOXA1 mutations contribute to the establishment of an immune-suppressive tumor microenvironment, highlighting distinct avenues for therapeutic intervention in FOXA1-mutant PCa tumors.

## Results

### FKHD missense mutations activate EMT pathway in vivo

**Fig. 1:**
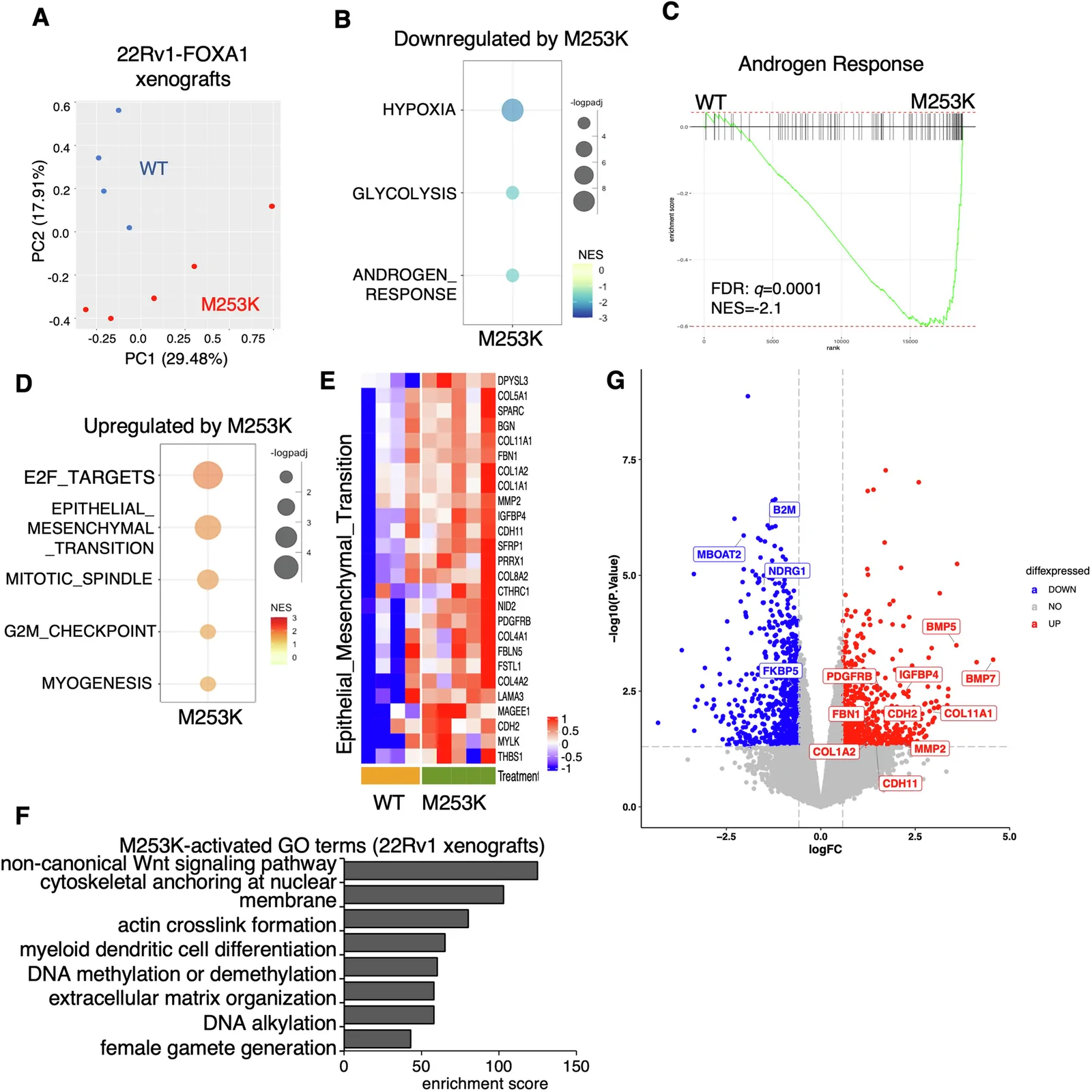
Transcriptomic analyses of 22Rv1-M253K xenograft tumors. 22Rv1 FOXA1 mutant xenograft samples were subjected to RNA-seq analysis. **A** Principal Component Analysis (PCA) of RNA-seq data (WT, *n* = 4; M253K, *n* = 5). **B**–**E** GSEA was performed by comparing M253K with WT for top ranked pathways that are downregulated (**B**, **C**) and upregulated (**D**) in M253K tumors. The heatmap view of significantly upregulated EMT genes (**E**) was shown. NES: Normalized Enrichment Score. **F** Gene ontology analysis of top ranked pathways that are upregulated by M253K xenograft tumors. **G** Volcano plot showing genes that were significantly up-and down-regulated in 22Rv1-M253K.

While recent studies have begun to uncover the molecular distinctions among different FOXA1 mutants, their phenotypic consequences remain poorly understood. Notably, FKHD mutants exhibit comparable transcript levels to the *FOXA1* amplification cases in patient samples. Our previous studies using 22Rv1 xenografts demonstrated that FOXA1-M253K confers a growth advantage over WT overexpression in subcutaneous tumor models^7^. To investigate the gene programs underlying this phenotype, we performed RNA-seq analysis on tumors from vehicle-treated mice (WT, *n* = 4; M253K, *n* = 5, all received doxycycline-containing chow). This analysis revealed distinct transcriptomic profiles between two groups (Fig. 1A). Gene Set Enrichment Analysis (GSEA) showed that classical AR signaling was negatively enriched in M253K tumors (Fig. 1B, C), consistent with previous observations in stable LNCaP cell lines^7^. Interestingly, the hypoxia pathway was also significantly downregulated in M253K tumors, suggesting that the mutants may influence the tumor microenvironment. In contrast, several proliferative pathways, including E2F targets and Mitotic spindle, were significantly enriched in M253K tumors, aligning with the observed growth advantage in our prior xenograft studies^7^. Furthermore, the EMT pathway was also enriched in M253K tumors, mirroring findings from LNCaP-based models (Fig. 1D, E). Gene Ontology analysis of significantly upregulated genes (log₂FC > 1, *p* < 0.05) identified pathways such as non-canonical Wnt signaling, actin crosslink formation, and extracellular matrix organization (Fig. 1F), implicating FOXA1-M253K in promoting cancer stemness, cell mobility, and metastasis. The findings in our 22Rv1-M253K subcutaneous tumors models are also consistent with previous mouse organoid studies^4^, where a subset of mutations drive EMT pathway.

The volcano plot in Fig. 1G highlights key transcriptomic changes induced by the M253K mutation, including activation of pro-metastatic genes and repression of androgen-responsive genes. Among the pro-metastatic genes upregulated by M253K, we observed members of the cadherin family (*CDH2*, *CDH11*), collagen family (*COL11A1*, *COL1A2*), and the extracellular matrix metalloproteinase *MMP2*—all of which are associated with enhanced cell motility and invasive potential. Additionally, activation of members of the non-canonical Wnt signaling pathway, including bone morphogenetic factors *BMP5* and *BMP7* suggested that M253K may also modulate developmental and stemness-related pathways. In contrast, classical AR/AR-V7 target genes such as *FKBP5* and *MBOAT2* were significantly downregulated, further supporting a suppression of classical AR signaling in M253K tumors. Together, these in vivo transcriptomic analyses reinforce the notion that FOXA1-M253K reprograms PCa cells toward a less androgen-dependent, more mesenchymal and metastatic phenotype.

### FKHD mutations activate HPSE and promote invasive phenotypes

**Fig. 2:**
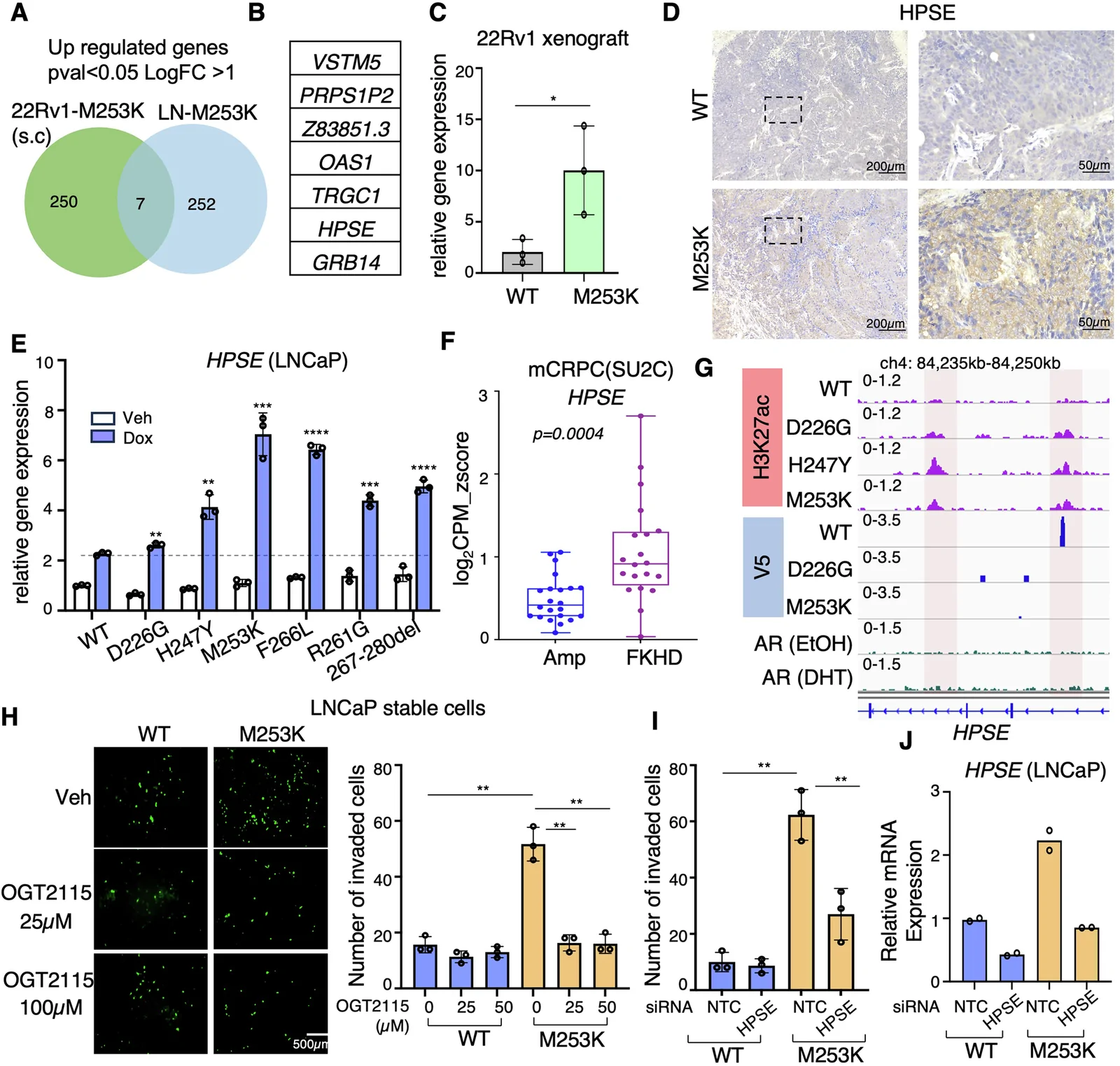
FKHD mutants upregulate HPSE expression. **A** Venn diagram of genes upregulated by M253K in comparison to WT from subcutaneous (s.c) xenograft tumors and LNCaP stable cells. **B** List of common genes identified from (**A**). **C**, **D** Expression of HPSE was measured by qRT-PCR (**C**, n = 3 independent samples) and IHC staining (**D**) in 22Rv1 s.c xenograft tumors. **E** Expression of HPSE in LNCaP stable cells (n = 3 independent samples). **F** Boxplot showing HPSE in CRPC patients (SU2C cohort) carrying different types of FOXA1 alterations (center: median; box: 25th to 75th IQR; whiskers: 1.5x IQR; statistical significance determined by unpaired two-sided t-test). **G** Genome browser view of FKHD-MS V5 ChIP-seq and H3K27ac peaks at *HPSE* locus. **H**–**J** Measurement of cell invasion. **H** Cells were treated with indicated inhibitors for 1 day. Representative images (left) and quantified results were shown (right). **I** Cells were treated with siHPSE and invaded cell were counted (n = 3 independent samples). **J** RT-qPCR of HPSE expression after knockdown (*n* = 2 independent samples). **C**, **E**, **H**, **I** data represented as mean ± SEM; statistical significance determined by unpaired two-sided t-test. *(0.01 < *P* < 0.05), **(0.001 < *P* < 0.01), ***(*P* < 0.001), and ****(*P* < 0.0001) were used to indicate the levels of *P*-value.

To identify target genes consistently activated by FKHD mutants, we compared the transcriptomic profiles of M253K-expressing LNCaP cells and 22Rv1 xenograft tumors. Using a threshold of log₂FC > 1 and *p* < 0.05, we identified seven overlapping genes, including several membrane-bound signaling molecules (Fig. 2A, B), underscoring the potential crosstalk between mutant cancer cells and the tumor microenvironment. Among these genes, several encode cell membrane proteins that may play roles in integrating extracellular signals. For example, *VSTM5* (V-Set and Transmembrane Domain Containing 5) is a putative cell adhesion protein and has been implicated in promoting neuronal migration^10^. *GRB14* (Growth Factor Receptor-Bound Protein 14) contains both a pleckstrin homology (PH) domain and a Src homology 2 (SH2) domain and is involved in insulin signaling and receptor tyrosine kinase pathways^11^.

HPSE, the only known mammalian endoglycosidase capable of degrading heparan sulfate, facilitates the release of growth-promoting and signaling molecules^12^. qRT-PCR analysis confirmed increased *HPSE* expression in M253K tumor samples (Fig. 2C). Immunohistochemistry (IHC) further validated a marked elevation of HPSE in the cytoplasm of M253K tumors (Fig. 2D). To assess whether *HPSE* is a common transcriptional target of FKHD alteration, we generated additional FOXA1 mutant cell lines using the doxycycline-inducible system. These include two FKHD hotspot missense mutants (R261G, F266L) and one in-frame deletion (ΔK267-S280) that produces a truncated Forkhead domain (Supplementary Fig. 1A, B). Similar to the previously studied missense mutations, these additional mutants impaired AR transcriptional activity and increased the expression of EMT genes, including *S100P*, *TGFB2* and *MET*. Moreover, these mutants lost binding on the canonical AR-regulated enhancers but gained binding on the EMT gene enhancers (Supplementary Fig. 1C–E). As shown in Fig. 2E, *HPSE* was consistently upregulated across various FKHD mutations, suggesting it is a common downstream target of FKHD mutants. To evaluate *HPSE* in mCRPC patients harboring FOXA1 alterations, we analyzed its enrichment in the SU2C cohort. Patients with FOXA1 alteration were stratified into two groups based on FOXA1 genetic status: WT-FOXA1 amplification (Amp) (*n* = 22), FKHD missense mutations/indels (*n* = 20). Compared to the amplification group, patients with FKHD mutations showed significant increase of *HPSE* (Fig. 2F).

We next analyzed the epigenomic landscape of the *HPSE* locus. In FKHD mutant cells, we observed activation of multiple intronic enhancers marked by increased H3K27ac levels (Fig. 2G). However, no concurrent increase in V5-FOXA1 binding was detected at these enhancers. In fact, mutant FOXA1 exhibited a loss of binding at one of these regions, indicating that additional mechanisms may contribute to HPSE upregulation. The heparanase inhibitor OGT 2115, previously shown to induce apoptosis in AR-negative PCa cells at IC50 values up to 100 µM^13^, also blocked the enhanced invasive capacity of M253K cells, without affecting WT cells and causing any cytotoxic effects in either cell lines (Fig. 2H**;** Supplementary Fig 2A). Similarly, knockdown of HPSE significantly reduced invasive capacity specifically in FKHD mutant cells, with minimal effect in WT controls (Fig. 2I, J), supporting a mutation-dependent functional role for HPSE in promoting invasive phenotypes. Moreover, prolonged treatment with OGT 2115 significantly impaired the growth of FKHD mutant cells, while having no observable effect on WT cells (Supplementary Fig. 2B). Overall, our findings identify HPSE as a transcriptional target commonly activated by FKHD mutants, with functional implications for tumor growth and invasiveness.

### FKHD mutations induce SE reprogramming

**Fig. 3:**
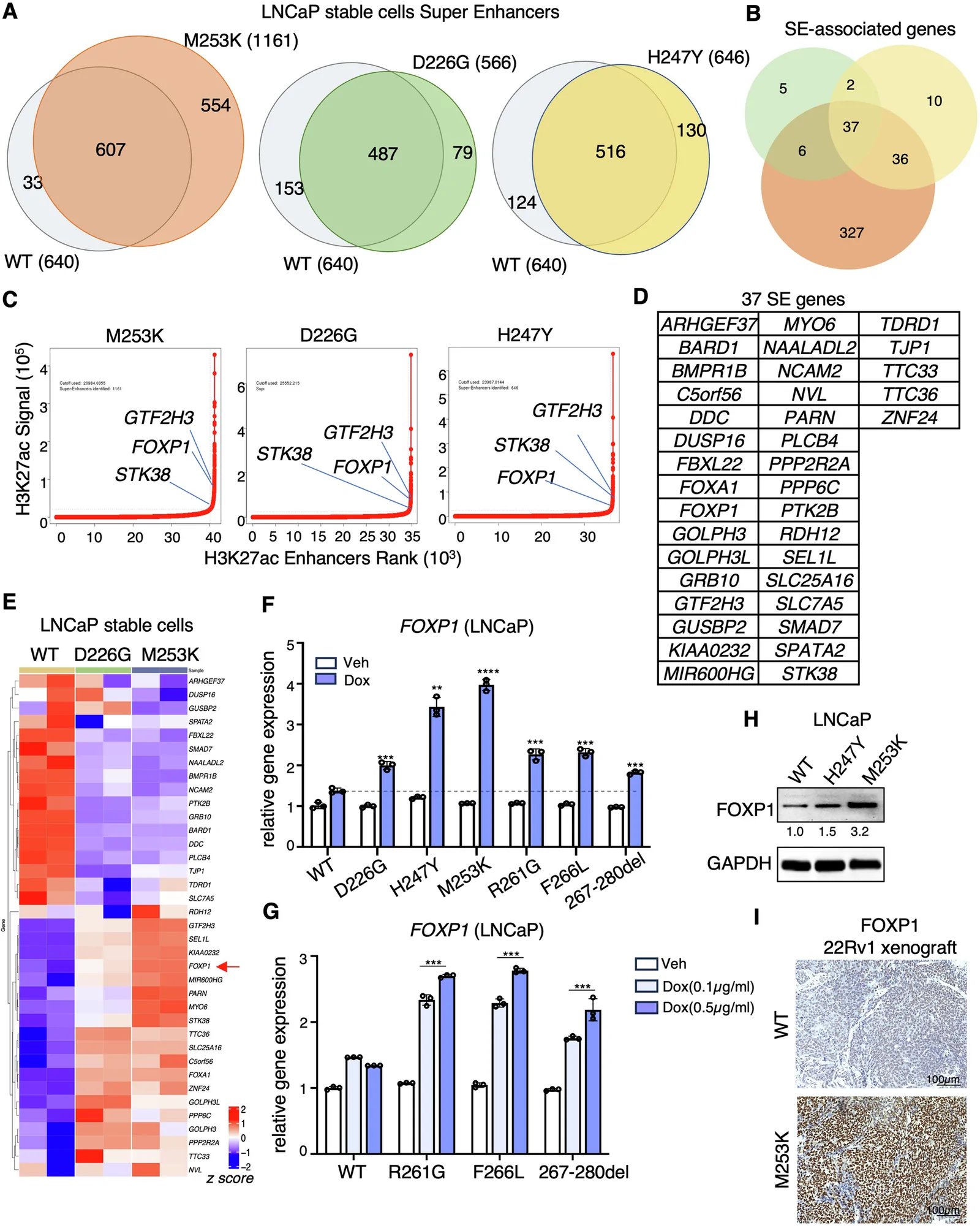
FKHD mutants drive super-enhancer (SE) reprogramming. **A** Number of SEs identified in different cell lines, and venn diagrams show the overlap comparing with WT cells. **B** Venn diagram of SEs-associated genes that are overlapped among different cell lines. **C** Top-ranked SE associated genes identified in mutant cells. **D** List of 37 SE-associated genes that are shared among three mutant cell lines. **E** Heatmap of the 37 SE genes expression in LNCaP overexpressing D226G and M253K. The gene levels were compared to WT in absence of DHT. **F**–**H** Expression of FOXP1 in LNCaP stable cells measured by qRT-PCR (**F**, **G**) (*n* = 3 independent samples; data represented as mean ± SEM; statistical significance determined by unpaired two-sided t-test) and western (**H**). **I** IHC staining of FOXP1 in 22Rv1 s.c xenograft tumors. *(0.01 < *P* < 0.05), **(0.001 < *P* < 0.01), ***(*P* < 0.001), and ****(*P* < 0.0001) were used to indicate the levels of P-value.

We previously reported that FOXA1 is enriched at SEs^14^. Based on this observation, we hypothesize that FOXA1 mutations may activate novel SEs programs that drive PCa progression. We previously have performed H3K27ac ChIP-seq studies in LNCaP cells expressing either WT or mutant FOXA1. Using the ROSE (Rank Ordering of Super-Enhancers) algorithm, we identified 640 SEs in the WT cell line. Each FOXA1 mutant cell line exhibited a distinct number of gained SEs, with the M253K mutation showing the most robust increase (Fig. 3A).

We next identified 37 genes that were consistently associated with these gained SEs across the mutant cell lines (Fig. 3B–D). Integrating our previously published RNA-seq data in LNCaP stable cells, we identified a subset of these SEs genes significantly upregulated by both D226G and M253K (Fig. 3E). Notably, these genes include *GTF2H3* (General Transcription Factor IIH Subunit 3), which interacts with CDK-activating kinase complex, and *STK38* (Serine/Threonine Kinase 38), a regulator of the cell cycle and apoptosis. Intriguingly, *FOXP1*, another pioneer factor, emerged as the top-ranked consistently upregulated across the mutants. Given its established role in promoting tumor aggressiveness in other malignancies, we further explored the regulation of FOXP1 by mutant FOXA1. Overexpression of these FOXA1 mutants consistently upregulated FOXP1 expression in the LNCaP stable cells (Fig. 3F–H). Although *FOXP1* mRNA showed a modest increase in the 22Rv1 mutant xenografts (Supplementary Fig. 3A), IHC of FOXP1 revealed a noticeable stronger staining in mutant tumor (Fig. 3I), supporting increased FOXP1 protein expression in vivo. However, knockdown of FOXP1 did not appear to reduce the invasive capacity of FKHD mutant cells (Supplementary Fig. 3B, C), suggesting a distinct functional contribution of FOXP1 in the mutant cells.

### FKHD mutations activate FOXP1 SE

**Fig. 4:**
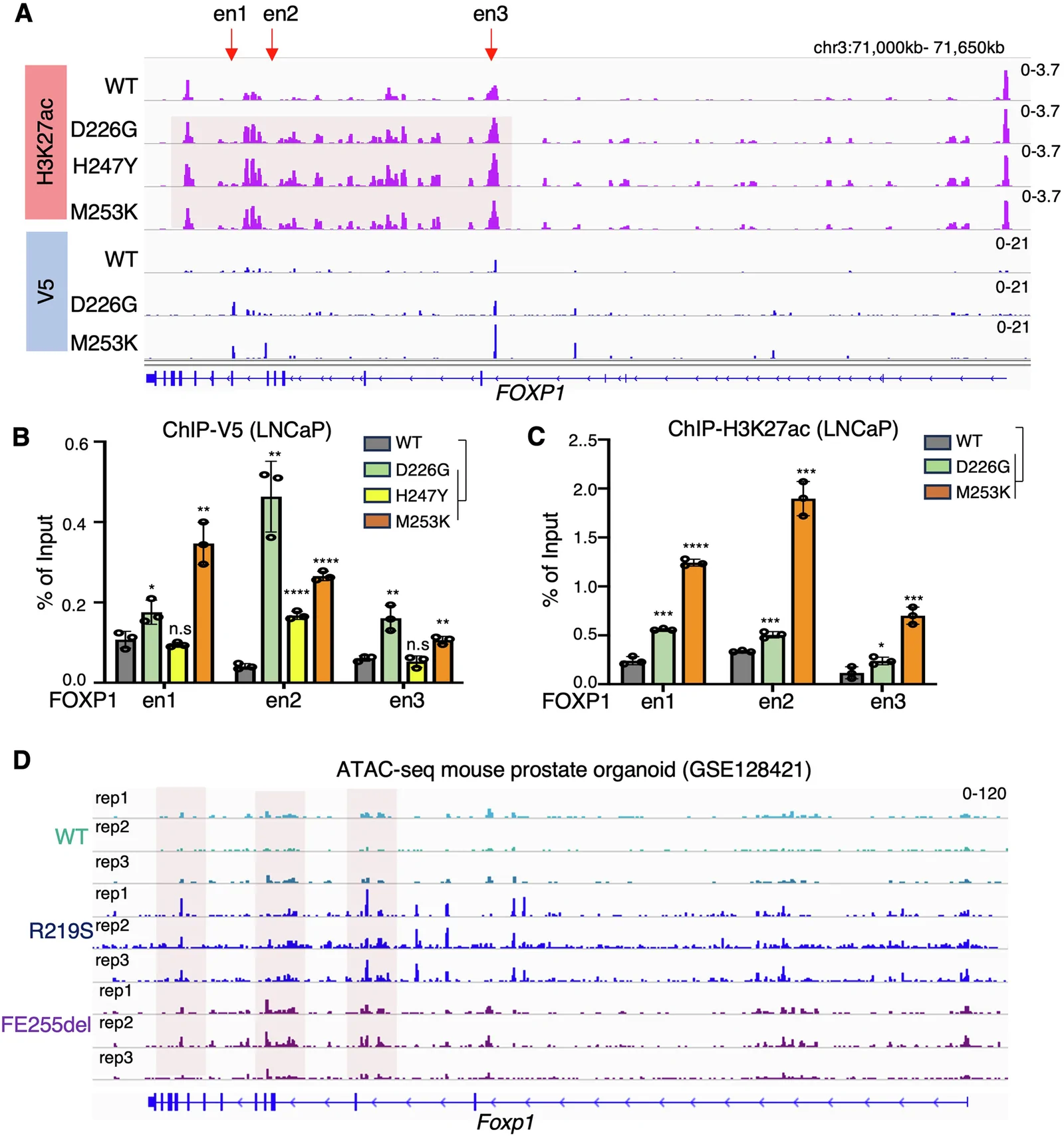
Epigenomic analyses of FOXP1 SE. **A** Genome browser view of ChIP-seq peaks at human *FOXP1* locus. SE is highlighted in shaded area. **B**, **C** ChIP-qPCR of V5 binding (B) and H3K27ac (**C**) at the indicated enhancers (n = 3 independent samples; data represented as mean ± SEM; statistical significance determined by unpaired two-sided t-test). **D** Genome browser view of ATAC peaks at mouse *Foxp1* locus in prostate organoids expressing different mutants. Gained accessible chromatin regions are highlighted. ns (*P* > 0.05), *(0.01 < *P* < 0.05), **(0.001 < *P* < 0.01), ***(*P* < 0.001), and ****(*P* < 0.0001) were used to indicate the levels of P-value.

We then focused on the SE regulating FOXP1 that is activated by FKHD mutants. As shown in Fig. 4A, we identified a SE within the *FOXP1* locus that was specifically activated in mutant cell lines. Concordantly, mutant FOXA1 exhibited increased binding within this SE. To determine whether enhanced chromatin occupancy by FKHD mutants leads to SE activation, we performed ChIP-qPCR using primers targeting three gained V5-FOXA1 binding peaks. The results confirmed stronger occupancy by the mutants and increased H3K27ac signal at these regions compared to WT FOXA1 (Fig. 4B, C). To further validate *FOXP1* SEs activation, we examined the public ATAC-seq datasets from mouse prostate organoids carrying FKHD mutants^4^. As shown in Fig. 4D, both missense and indel mutants showed broad increase of chromatin accessibility within the *Foxp1* locus. Collectively, these findings indicate that FOXA1 FKHD mutants activate a SE that enhances FOXP1 expression, which may promote mutant-driven PCa progression.

**Fig. 5:**
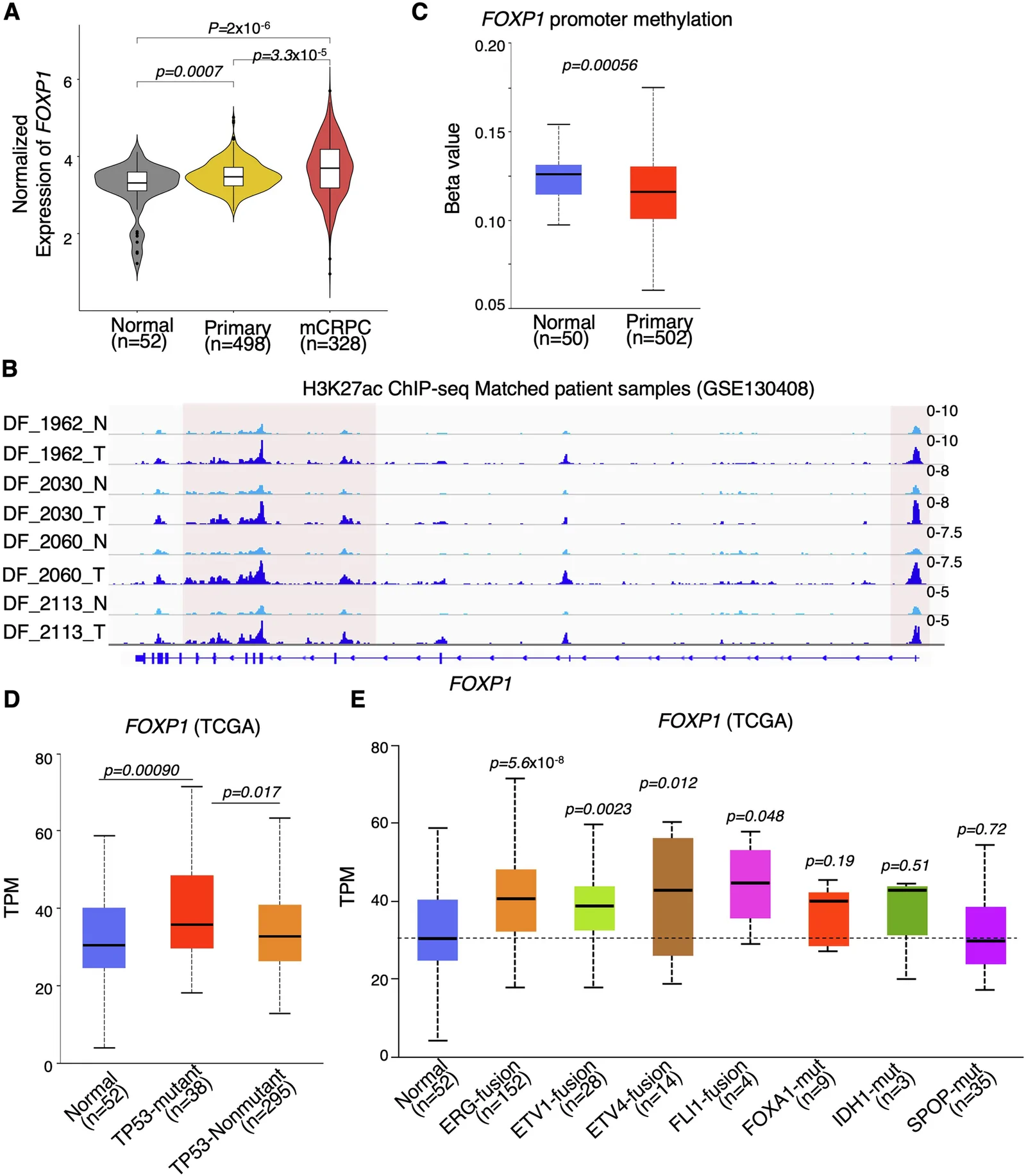
Expression of FOXP1 in clinical samples. **A** Violin plot of *FOXP1* expression in PCa patients of different stages (center: median; box: 25th to 75th IQR; whiskers: 1.5x IQR; shape: kernel density estimate, statistical significance determined by unpaired two-sided t-test). **B** Genome browser view of H3K27ac ChIP-seq peaks at *FOXP1* locus in matched PCa patient samples. **C** The methylation levels of *FOXP1* promoter in PCa patients (TCGA cohort). **D**, **E** *FOXP1* expression levels in TCGA patients with distinct molecular features. Statistical significance was determined by comparing to normal samples. C-E: center: median; box: 25th to 75th IQR; whiskers: 1.5x IQR; statistical significance determined by unpaired two-sided t-test.

We next examined *FOXP1* expression in multiple public PCa patient datasets. *FOXP1* was significantly increased in CRPC in comparison with primary PCa and normal prostate samples (Fig. 5A), suggesting a potential role in disease progression. Interestingly, in a public PCa dataset with matched normal and tumor tissues^15^, H3K27ac ChIP-seq revealed substantially stronger signals at the *FOXP1* SEs in tumor samples compared to their matched normal tissues (Fig. 5B), consistent with elevated *FOXP1* expression in tumors. Additionally, the elevated *FOXP1* expression may also be attributed to decreased promoter methylation (Fig. 5C). Recent reports have shown that FKHD Wing2 mutation events (defined as Class 1) frequently co-occur with biallelic TP53 loss, and transgenic mice harboring both alterations develop high-grade metastatic PCa^9^. Notably, using a web sourced tool UALCAN^16^, we observed that primary PCa patients with TP53 mutations exhibited significantly higher *FOXP1* expression compared to those with WT TP53 (Fig. 5D). *FOXP1* expression was broadly elevated across patients with diverse molecular subtypes, although its levels were modestly increased in tumors harboring FOXA1 mutations (Fig. 5E). Collectively, these findings suggest that FOXP1 is a consistently upregulated, SE–associated transcriptional target particularly in the context of FKHD mutants.

### FKHD mutations associate with immunosuppressive microenvironment

**Fig. 6:**
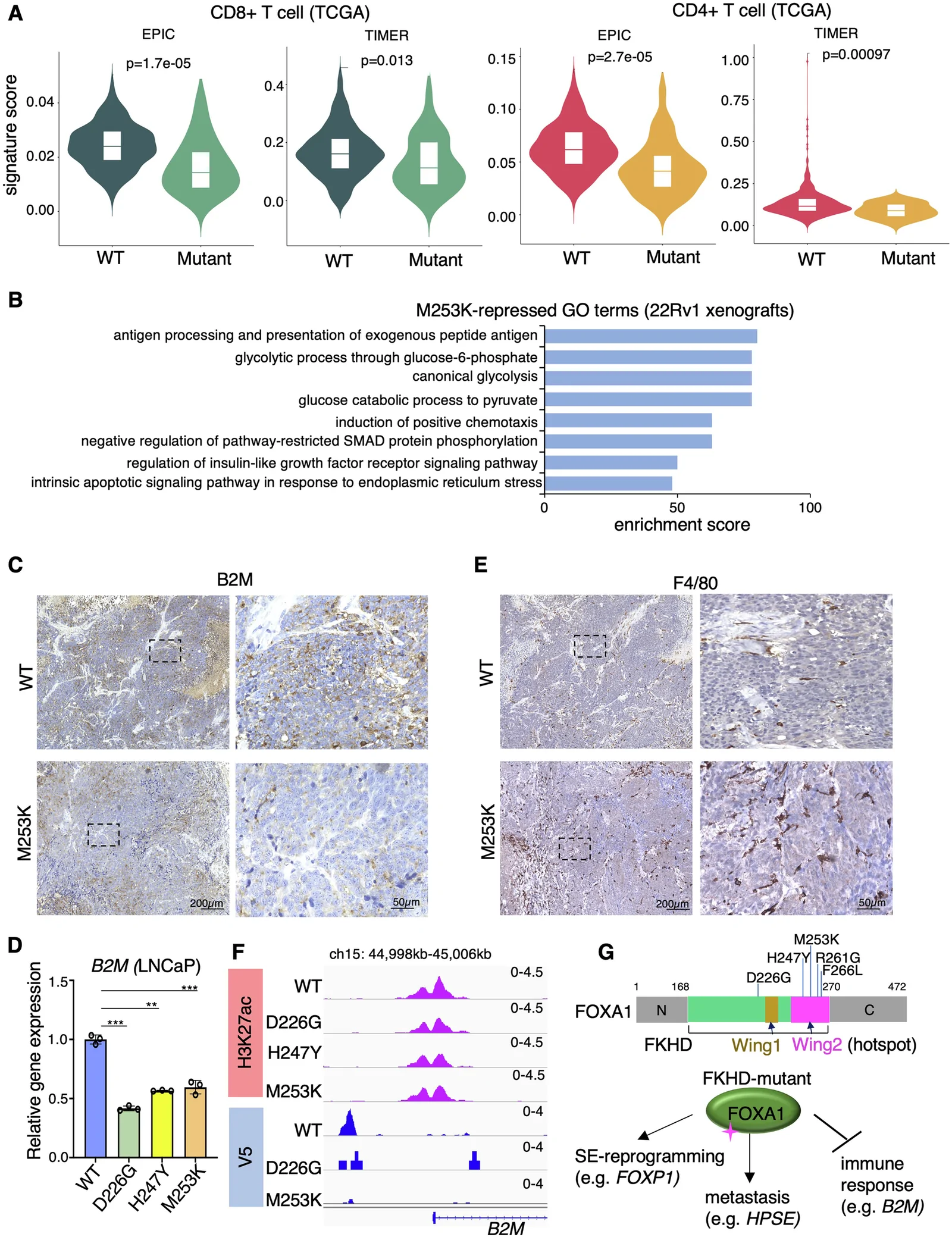
FKHD mutations associate with immunosuppressive microenvironment. **A** Violin plot of immune infiltrates signatures using indicated algorithm in primary PCa patients (TCGA cohort) (center: median; box: 25th to 75th IQR; shape: kernel density estimate, statistical significance determined by unpaired two-sided t-test). **B** Bar plot of the top-ranked pathways that were downregulated in 22Rv1-M253K subcutaneous tumors. **C** IHC staining of B2M in the 22Rv1 subcutaneous xenografts. **D** qRT-PCR analyses for *B2M* in FKHD-MS stable cells (*n* = 3 independent samples; data represented as mean ± SEM; statistical significance determined by unpaired two-sided t-test). **E** IHC staining of F4/80. **F** Genome browser view of ChIP-seq peaks at *B2M* enhancer. **G** Summary of FKHD mutant functions. **(0.001 < *P* < 0.01), ***(*P* < 0.001), were used to indicate the levels of P-value.

PCa typically exhibits an immunosuppressive tumor microenvironment. However, the role of FOXA1 mutations in modulating immune responses in PCa remains unclear. To explore this, we employed computational immune deconvolution methods to estimate immune cell infiltration from bulk tumor transcriptome data. Using EPIC (Estimating the Proportions of Immune and Cancer cells)^17^ and TIMER2.0 (Tumor Immune Estimation Resource)^18^, we examined the association between FOXA1 mutations and immune cell infiltration. In primary PCa tumors from TCGA cohort, tumors harboring FOXA1 mutations showed significantly reduced signatures of CD4⁺ and CD8⁺ T cells (Fig. 6A). However, no significant differences in immune cell abundance were detected between FOXA1-mutant and wild-type tumors in mCRPC patients (supplementary Fig. 4). This suggests that FOXA1 mutations may not be the primary drivers of immune suppression in metastatic disease. This observation aligns with recent findings showing that FOXA1 overexpression can repress interferon signaling by physically blocking STAT2 binding to chromatin, an effect independent of its transactivation function or mutational status^19^. Nonetheless, based on transcriptomic profiles from tumors expressing the FKHD domain mutant M253K, we hypothesized that FOXA1 mutations may still transcriptionally repress immune-related pathways. Indeed, gene set enrichment analysis revealed that the most significantly downregulated programs in 22Rv1 M253K xenograft tumors were associated with antigen processing and presentation (Fig. 6B). Among these, we observed marked downregulation of B2M (Beta-2 Microglobulin), a critical component of MHC class I molecules, in M253K tumors. Consistent with this, IHC analysis showed reduced membrane localization of B2M in M253K tumors (Fig. 6C), suggesting a more immunosuppressive microenvironment. Interestingly, B2M levels were also decreased in LNCaP cell lines stably expressing the FKHD mutants (Fig. 6D).

Although the xenograft tumors were developed in SCID mice (which lack functional T and B cells), innate immune cells such as macrophages and NK cells remain present. We therefore hypothesized that M253K tumors might promote increased infiltration of tumor-supportive immune cells or decreased presence of tumor-suppressive immune populations. Surprisingly, we found a marked increase in F4/80⁺ macrophages, a well-established marker of M2-like tumor-associated macrophages (TAMs), in M253K tumors (Fig. 6E), suggesting that the mutant tumor microenvironment contributes to macrophage recruitment or polarization.

To explore the mechanism underlying B2M downregulation, we analyzed our V5-FOXA1 ChIP-seq data from LNCaP stable cell lines. We observed that FOXA1 mutants exhibited reduced binding at a putative enhancer region near the *B2M* locus, along with decreased H3K27ac at the B2M promoter (Fig. 6F), indicating a loss of enhancer activity and transcriptional repression. Together, these findings suggest that FOXA1 mutations can transcriptionally suppress key immune response genes, such as *B2M*, and reshape the tumor immune microenvironment, potentially by dampening antigen presentation and altering macrophage infiltration.

## Disscussion

Our transcriptomic profiling of FKHD mutant xenograft tumors revealed Hallmark pathway alterations similar to those observed in our previously characterized LNCaP stable cell line models. These findings support that FKHD mutations, including Wing2 missense variants and in-frame deletions, diminish canonical AR activity while activating EMT pathways. We also found that FKHD mutant may play a role in facilitating PCa cell immune evasion. FOXA1 FKHD mutations are known to alter chromatin binding specificity, chromatin mobility, and enhancer selection. We suggest that these biophysical and epigenomic changes create a permissive chromatin environment that allows distinct downstream programs—such as EMT activation, super-enhancer reprogramming (e.g., FOXP1), and repression of antigen presentation genes (e.g., B2M)—to be engaged in a locus- and context-specific manner (summarized in Fig. 6G). Importantly, these programs are unified by their dependence on mutant FOXA1-driven chromatin reorganization, even if they do not directly regulate one another.

Notably, a recent large-scale sequencing study reported that distinct FOXA1 mutations are generally associated with increased AR signaling and reduced EMT pathway activity^2^, an observation seemingly contradictory to our results. However, it is important to note that their conclusions were drawn from comparisons between mutant and WT samples, where WT FOXA1 expression levels were markedly lower than those in the mutant group. In our studies, by contrast, WT FOXA1 was overexpressed, modeling the gene amplification (class 4) characteristic of FOXA1 alterations. This distinction in reference conditions may account for the contrasting pathway activity profiles observed. A previous report using stable cell line models offered additional insights into how Wing 2 mutations can promote AR signaling, specifically through increased chromatin mobility and chromatin sampling frequency (“fast”)^3^. More recently, the same group demonstrated in transgenic mouse models with compromised Wing 2 (R265-Q271del) can lead to extensive reprogramming of the AR cistrome^9^. Notably, this redistribution of AR binding appears to be dependent on the NSD2 methyltransferase. Together, both studies suggest that Wing 2 mutation-mediated AR activity is critical for reinforcing the luminal features in prostate adenocarcinoma.

Interestingly, we also observed that pathways associated with the Warburg effect, such as glycolysis and hypoxia, were significantly downregulated in M253K xenograft tumors. FOXA1 has been shown to transcriptionally repress the hypoxia program by directly downregulating HIF1A expression^20^. In comparison to FOXA1 overexpression, our data suggest that the mutant FOXA1 may shift the tumor microenvironment from hypoxia toward normoxia, thereby facilitating cancer cell survival and metastasis. These findings also raise the possibility that metabolic reprogramming may contribute to immune suppression in FOXA1-mutant PCa, which requires future investigations. Although hypoxia is well known to drive EMT and promote invasion^21^—seemingly inconsistent with the primary function of FKHD mutants, this paradox may be reconciled by previous findings that hypoxia enhances AR transcriptional activity^22^. Thus, repression of hypoxia by FKHD mutants may also contribute to the observed attenuation of classical AR signaling. Nonetheless, we do not exclude the possibility that certain metabolites resulting from this metabolic shift may also confer selective advantages to mutant cells.

Although the molecular mechanisms underlying the activity of various FOXA1 mutants, such as altered chromatin occupancy, have been elucidated, effectively targeting mutant cells remains a major challenge. Through systematic transcriptomic profiling, our study identifies HPSE as a notable downstream target of FKHD domain mutants. HPSE is an endoglycosidase that degrades heparan sulfate (HS) chains on proteoglycans, thereby remodeling the extracellular matrix (ECM). This enzymatic activity facilitates the release of HS-bound signaling molecules, including mitogens, and contributes to key oncogenic processes such as cell migration and invasion. Overexpression of HPSE has been reported across a wide range of cancers and is closely associated with tumor metastasis and angiogenesis^12,23,24^.

Due to its critical role in tumor progression, HPSE has emerged as an attractive therapeutic target. Several strategies have been developed to inhibit HPSE activity, including enzymatic inhibitors and heparan sulfate mimetics. For instance, cyclophellitol-derived HPSE inhibitors have recently demonstrated efficacy in reducing tumor metastasis^25^. Among HS mimetics, SST0001 (Roneparstat) has progressed to a Phase I clinical trial for refractory multiple myeloma (NCT01764880), while PG545 (pixatimod) has entered clinical evaluation for advanced solid malignancies (Phase I, NCT02042781). PI-88 (muparfostat) was assessed as adjuvant therapy in primary liver cancer (Phase III NCT01402908), and M402 (Necuparanib) has been investigated for metastatic pancreatic cancer in combination with nab-paclitaxel and gemcitabine (Phase I/II NCT01621243). However, the limited commercial availability of these inhibitors currently precludes direct testing in our FOXA1-mutant PCa models. Regardless, our findings suggest that HPSE is a promising biomarker in PCa patients harboring FKHD domain mutations. Targeting HPSE may offer a distinct therapeutic avenue for this genetically defined subset of patients.

Mechanistically, in contrast to EMT-related genes (e.g., *TGFB2*, *MET*), which exhibited enhanced FKHD mutant binding at their enhancers, the *HPSE* enhancer showed reduced FKHD binding. This observation is similar to the recent study showing loss of FOXA1 mutant binding on its target SEMA3C^8^. These effects suggest that FKHD mutants may interact with unknown transcriptional repressors. It is also possible that FKHD mutants reshape the epigenetic landscape of FOXA1 by altering its interactions with chromatin regulators. Among these, LSD1 has been identified as a critical factor for enhancer activation, particularly at SEs^14^, while EZH2 has been implicated in driving EMT in enzalutamide-resistant PCa^26^. Interestingly, both histone modifiers have been reported to influence the methylation status of FOXA1^27,28^. However, whether FKHD mutations affect FOXA1’s interaction with these epigenetic partners remains to be determined.

Aberrant enhancer activation is a key driver of oncogenic transcriptional reprogramming during cancer progression. Among the top SE-associated genes, *FOXP1* emerged as a prominent target. Notably, FOXP1 has been reported to interact with NFAT1 to enhance breast cancer cell motility^29^. However, in our system, FOXP1 depletion did not significantly affect the invasive phenotype of FOXA1 FKHD mutant cells, suggesting that FOXP1 is not a major mediator of this particular phenotype. Importantly, prior studies have established critical roles for FOXP proteins in suppressing immune responses in immune cells, including T cells, B cells, and macrophages^30–33^. Given these well-documented immunoregulatory functions, we cannot exclude the possibility that FOXP1 upregulation in FOXA1 FKHD mutant tumor cells may indirectly contribute to immune modulation, for example by influencing cytokine expression, immune signaling pathways, or tumor–immune interactions. Although *FOXP1* transcript expression was not significantly increased in FOXA1-mutant patient samples, multiple factors including cofactor usage, genetic background, and tumor heterogeneity may contribute to this discrepancy, as suggested by our xenograft studies. In addition to *FOXP1*, we identified other SE-driven genes such as *GTF2H3* and *STK38*, which were upregulated specifically in FKHD mutant cells and may play essential, context-dependent roles in supporting the mutant phenotype. Notably, the mutants appear to also activate the *FOXA1* SE, which may explain the elevated mutant transcript levels observed in the patient samples.

FOXA1 has been previously reported to suppress interferon signaling and immune responses, contributing to an immunosuppressive tumor microenvironment in PCa^19^. Interestingly, this repressive function of FOXA1 is independent of its transcriptional activity; instead, FOXA1 directly binds to the DNA-binding domain of STAT2, blocking its ability to access chromatin. Interestingly, immune response genes were also commonly upregulated by M253K in different models. *OAS1* (2’-5’-Oligoadenylate Synthetase 1) is activated by double-stranded RNA and exhibits antiviral activity. A pan-cancer study has shown that elevated OAS1 expression is associated with poor prognosis across multiple malignancies, including PCa^34^. Overexpression of OAS1 may enhance cancer cell survival under conditions of DNA damage^35^. Another gene, *TRGC1* (T Cell Receptor Gamma Constant 1), encodes a component of the T-cell receptor and has been proposed as a biomarker of poor prognosis in breast cancer^36^. Our findings suggest that FOXA1 mutations may further modulate the tumor immune microenvironment, adding another layer of complexity to its role in immune regulation.

In summary, our RNA-seq analyses of FKHD mutant subcutaneous xenograft tumors revealed several actionable downstream targets that may be therapeutically exploited in PCa patients harboring these mutations. Although EMT activation, SE reprogramming, and immune suppression appear mechanistically distinct, these pathways are unified by their dependence on altered FOXA1 FKHD function. FKHD mutations likely rewire the chromatin landscape by modifying FOXA1 DNA-binding dynamics, chromatin mobility, and enhancer selection. This global reprogramming permits the activation or repression of discrete transcriptional programs in a context-dependent manner.

## Methods

### Cell culture

CWR-22RV1 (22RV1) and LNCaP cell lines were purchased from ATCC, authenticated every 6 months using short tandem repeat profiling, and frequently tested for Mycoplasma contamination using the MycoAlert Mycoplasma Detection Kit (Lonza). 22RV1, LNCaP, and LNCaP-derived stable cell lines were cultured in RPMI-1640 medium with 10% FBS, which was replaced by the hormone-depleted medium (5% CSS) for 2–3 days before the subsequent assays. ON-TARGETplus HPSE, FOXP1 SMARTpool siRNA (Horizon Discovery) was used to knockdown *HPSE* and *FOXAP1* respectively in the stable cells.

### Xenograft tumors

The mouse xenograft tumors expressing WT or M253K FOXA1 were collected from previous animal studies^7^. Mouse experiments were conducted in accordance with institutional and U.S. national guidelines and were approved by the IACUC of University of Massachusetts Boston. Male SCID mice (~6 week-old) were purchased directly from Taconic and housed in shoebox cages with solid floors covered with bedding materials. At the designated time points, tumor length (L) and width (W) were measured with calipers, and tumor volume was calculated using the formula L × W² / 2. According to the approved IACUC protocol, the maximum allowable tumor size was 1.5 cm in any dimension at any time, and this limit was not exceeded in any experiment. Animals were euthanized in accordance with institutional guidelines using an automated CO_2_ delivery system. The system uses a controlled three-stage process consisting of CO_2_ charging, dwell time, and evacuation, and the duration of exposure was sufficient to ensure death. All animal experiments were conducted in compliance with relevant ethical regulations for animal research.

### Cell invasion assay

Corning™ BioCoat™ Matrigel™ Invasion Chamber with Corning™ Matrigel Matrix transwell inserts placed in a 24-well plate (354480, Corning) containing cell culture medium. A total of 2 × 10^4^ cells were seeded in the upper chamber of the inserts with serum-free medium, while the lower chamber was filled with medium containing 20% serum. The cells were then cultured for 36–48 h at 37 °C with 5% CO_2_. Subsequently, the invading cells in the lower chamber were stained by Corning CalceinAM Fluorescent Dye (354217, Corning). All experiments were done in biological triplicates and images were taken by the Keyence Microscope.

### Cell proliferation assay

Cells were seeded in 96-well plates and treated with inhibitors for 4 days. Cell viability was measured using CellTiter-Glo Luminescent Cell Viability Assay (Promega) per the manufacturer’s protocol. The luminescence was detected on Omega plate reader by BMG LABTECH.

### Total RNA extraction, qRT-PCR, and RNA-Seq analysis

TRIzol reagent (Invitrogen) was used for RNA extraction according to the manufacturer’s protocol. qRT-PCR was performed using Taqman probe/primer mixes and Fast 1-step Mix (Thermo Fisher Scientific) on QuantStudio 6 pro PCR machine. The qRT-PCR results were normalized with *GAPDH* and quantified using the ΔΔCt method. The TaqMan primers and probes were purchased as pre-designed mixes from Thermo Fisher Scientific: *HPSE* (Hs00935036_m1), *FOXP1* (Hs00908900_m1), *B2M* (Hs00187842_m1).

RNeasy Kit (Qiagen) was used for tumor RNA extraction per manufacture’s instruction. For RNA-seq, The TruSeq Strand Total RNA LT kit (Illumina) was used for library preparation. Illumina NextSeq 2000 (51nt, pair-end) were used for next generation sequencing. The human reference genome (hg19) was used to align transcriptome-sequencing reads by using STAR (version 2.7.1a). featureCounts (version 2.0.1) from GRCh37 Ensembl reference was used for counting. R package Edger (3.36.0) was then employed to process all gene counts and evaluate the differential expression by using the Benjamini-Hochberg false discovery rate (FDR)-adjusted *P*-value. The expression values were normalized by centering and scaling across samples and displayed using the ComplexHeatmap (version 2.10.0) R package. Gene Set Enrichment Analysis (GSEA) was performed using Software GSEA (version 4.2.2) and R package fgsea (version 1.20.0).

### Patient data and publicly available datasets

UALCAN (http://ualcan.path.uab.edu/index.html), a web-based tool for analyzing TCGA RNA-seq and clinical data, was used to evaluate the expression of *FOXP1* in PCa patients of different molecular features^16^. For Immune Cell Estimation, Immune cell types can be inferred using the deconvolution-based approaches, TIMER 2.0^18^ and EPIC^17^ from R package immunedeconv^37^ (2.0.4). These approaches quantify the proportions of different immune cell types present in the heterogeneous SU2C patient samples via the deconvolution of bulk RNA sequencing data.

### ChIP (Chromatin Immunoprecipitation assay)

ChIP assay was performed based on the previously described protocol^28^. Briefly, LNCaP-FKHD-MS stable cells after treatment of doxycycline were fixed with 1% formaldehyde and lysed using ChIP lysis buffer (1% SDS, 5 mM EDTA, 50 mM Tris–HCl pH 8.1). Chromatin was then sheared to produce ~500–800 bp fragments using a Bioruptor Pico (Diagenode). Immunoprecipitation was carried out using anti-V5 (Thermo Fisher, catalog no. R960-25, 1:250), anti-H3K27ac antibody (Abcam, catalog no. ab4729, 1:1000). Precipitated protein-DNA complexes were reverse-crosslinked at 65 °C, followed by DNA purification using Qiagen QIAquick Purification Kit. The extracted DNA was then subjected to ChIP-qPCR using SYBR green on QuantStudio 6 pro real-time PCR machine. Primers for KLK3-ARE3, MET-FmBS were previously described^7^. Primers for sequences for FOXP1 super-enhancers: en1, Forward: 5’- GCCTCAGAAATTCCAAAATGACC-3’, Reverse: 5’-CACCATGTTCAAGTGTAAAACCA-3’; en2, Forward: 5’-TACTCGACTGTCTCTGCCTG-3’, Reverse: 5’- CAGGCCTCTTGTGTGTATGC-3’. en3, Forward: 5’-TGGGCCACATCACCTCAATA-3’, Reverse: 5’- ACACACACACACACACACAC-3’.

### Super-enhancer identification

H3K27ac ChIP-seq datasets used in this study were previously published (GSE133387)^7^ and were analyzed using the finalized signal tracks without additional normalization. We conducted SEs analyses as previously described^14^. The rank ordering of SEs (ROSE) algorithm^38,39^ was used to identify SEs using H3K27ac. ROSE was run by allowing enhancers within 12,500 bp to be stitched together. Stitched enhancers were ranked according to the intensity of the H3K27ac ChIP-seq signal. A threshold was then determined according to the geometric inflection point to distinguish between enhancers and SEs. The identified SE regions were further annotated by annotatePeaks. The overlap of SEs between FKHD mutants and WT were determined using bedtools.

### Immunoblotting

For the extraction of proteins, cells were lysed using 2% SDS and boiled for 5min. Protein samples were loaded onto 4 ~ 15% Mini-PROTEAN TGX precast protein gels (Bio-Rad) and transferred onto nitrocellulose membranes (Bio-Rad). The membranes were then blocked with 5% non-fat milk and incubated overnight at 4 °C with the following antibodies: anti-FOXP1 (CST 2005S, 1:500), anti-V5 (ThermoFisher R96025, 1:2000), anti-FOXA1 (Ab23738, 1:1000), and anti-GAPDH (CST 5174, 1:5000). Membranes were subsequently incubated with secondary antibodies labeled with fluorescence (ThermoFisher) or HRP (CST 7074) in 5% non-fat milk for 1 hr at room temperature. Gel images were captured using the iBright Imaging System (ThermoFisher).

### Immunohistochemistry staining

The staining was performed following the protocol listed on Cell Signaling Technology. Paraffin-embedded xenograft tissue sections were firstly deparaffinized and rehydrated through serial dilution of ethanol. Then, rehydrated sections underwent antigen retrieval by incubation in antigen unmasking solution (CST 14746) at 95 °C for 10 min, followed by cooling for 30 min. Sections were treated with 3% hydrogen peroxide solution and blocked with 10% Goat serum for 1 hr. Sections were then incubated with primary antibodies diluted in SignalStain Antibody Diluent (CST 8112) at 4 °C overnight. On next day, the sections were incubated with SignalStain Boost Detection Reagent (CST 8114) in a humidified chamber for 30 min at room temperature. The signals were detected using SignalStain DAB substrate Kit (CST 8059). DAB substrate was added to the slides, which were scanned by the Keyence Microscope. The primary antibodies include HPSE (ABclonal A16488, 1:250), FOXP1 (CST 4402S, 1:500), F4/80 (CST 70076, 1:500).

### Statistics and reproducibility

Data in bar graphs represent the mean ± SD of at least 3 biological repeats. For most studies, statistical analysis was performed using Student’s t-test by comparing treatment versus vehicle or otherwise as indicated: *p < 0.05; **p < 0.01; *** *p* < 0.001; ****p < 0.0001. No error bars or statistic *p*-values were presented or calculated for analysis with *n* = 2. All other statistical analyses and visualization were performed with R (version 4.2.0) unless otherwise specified.

### Reporting summary

Further information on research design is available in the Nature Portfolio Reporting Summary linked to this article.

## Supplementary information

Supplementary Information

Description of Additional Supplementary Files

Supplementary Data

Reporting Summary

## Data Availability

The RNA-seq data generated in this study have been deposited in the Gene Expression Omnibus (GEO) database under accession code GSE306036. All uncropped and unedited blot images are included in Supplementary Information (Supplementary Fig. 5). Source data can be found in the Supplementary Data. All other data are available from the corresponding author (or other sources, as applicable) on reasonable request.
