# Supplementary material for: Identification of targets of clinical FOXA1 mutants involved in EMT and immune suppression in prostate cancer
Source: Commun Biol. 2026 Jun 23;9:1326. doi: 10.1038/s42003-026-10523-2 (PMC13655023; doi:10.1038/s42003-026-10523-2)
Supplement: Supplementary file 1 [file 42003_2026_10523_MOESM1_ESM.pdf]

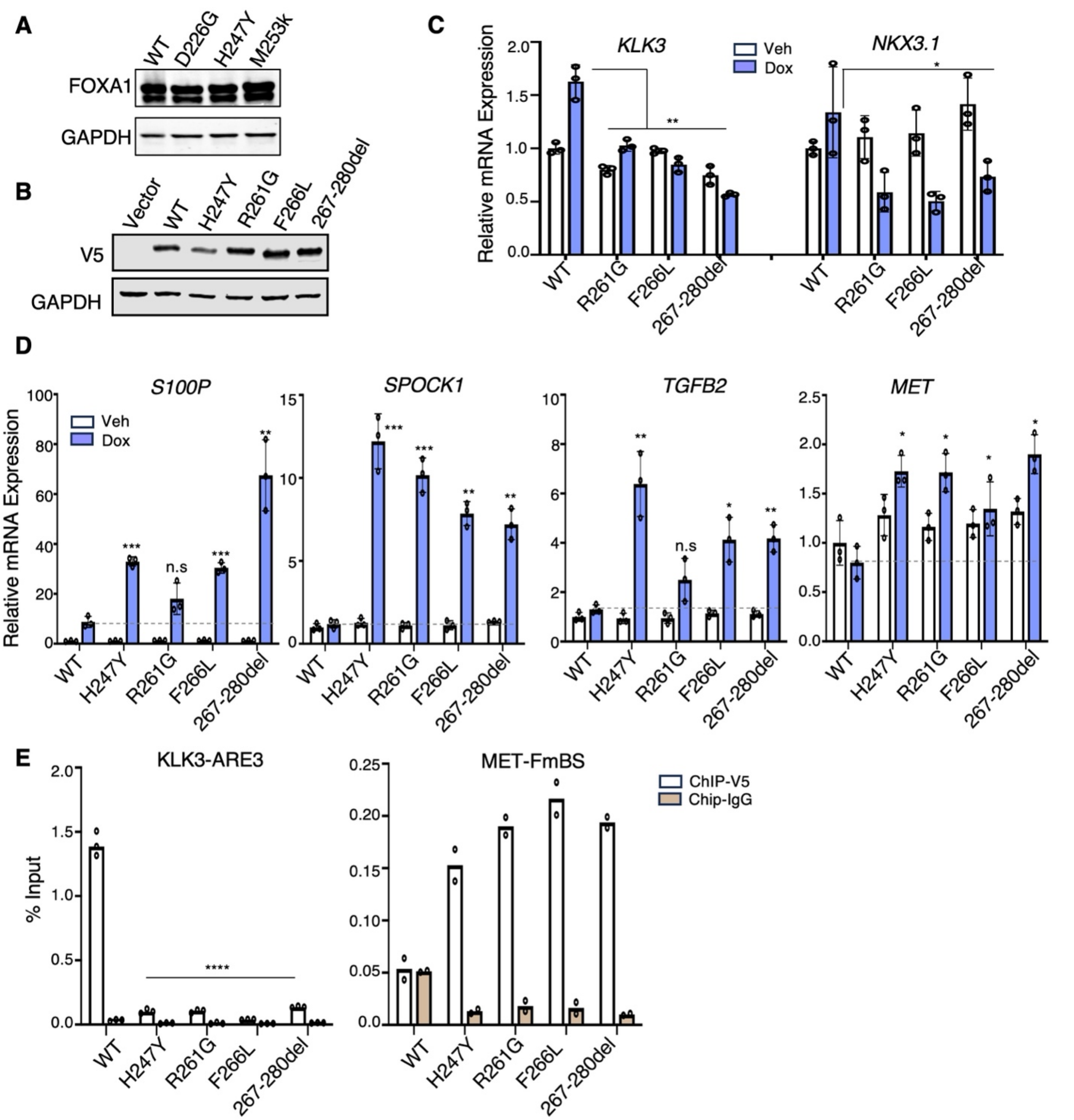

Supplementary Figure 1. Establishment of additional FKHD mutant cells in LNCaP. A, B Establishment of LNCaP stable cell lines expressing doxycycline-inducible V5-tagged FKHD-mutations. All conditions were treated with doxycycline for 2 days. **C** qRT-PCR of AR-activated genes in the mutant cell lines without treatment of DHT. **D** qRT-PCR of EMT genes in the mutant cell lines. **E** ChIP-V5 of FOXA1 mutant binding sites at KLK3 and MET enhancers. (C-E; n = 3

independent samples; data represented as mean ± SEM; statistical significance determined by unpaired two-sided t-test).

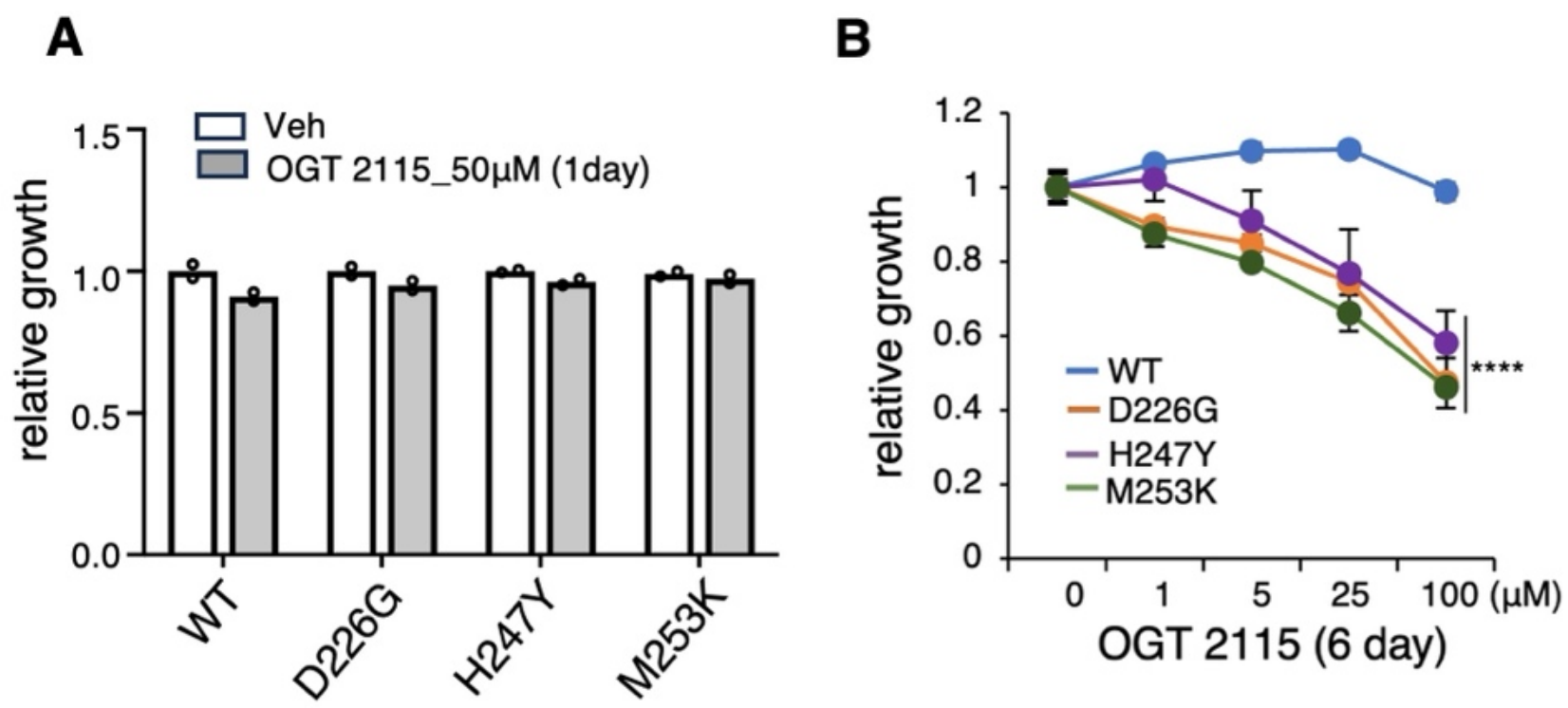

Supplementary Figure 2. OGT 2115 effects on FKHD mutant cell proliferation. Cell viability assay for LNCaP mutant stable cells treated with HPSE inhibitor OGT 2115 for 1day (A) (n = 2) and 6 days (B) (n = 3 independent samples; data represented as mean ± SEM; statistical significance determined by unpaired two-sided t-test).

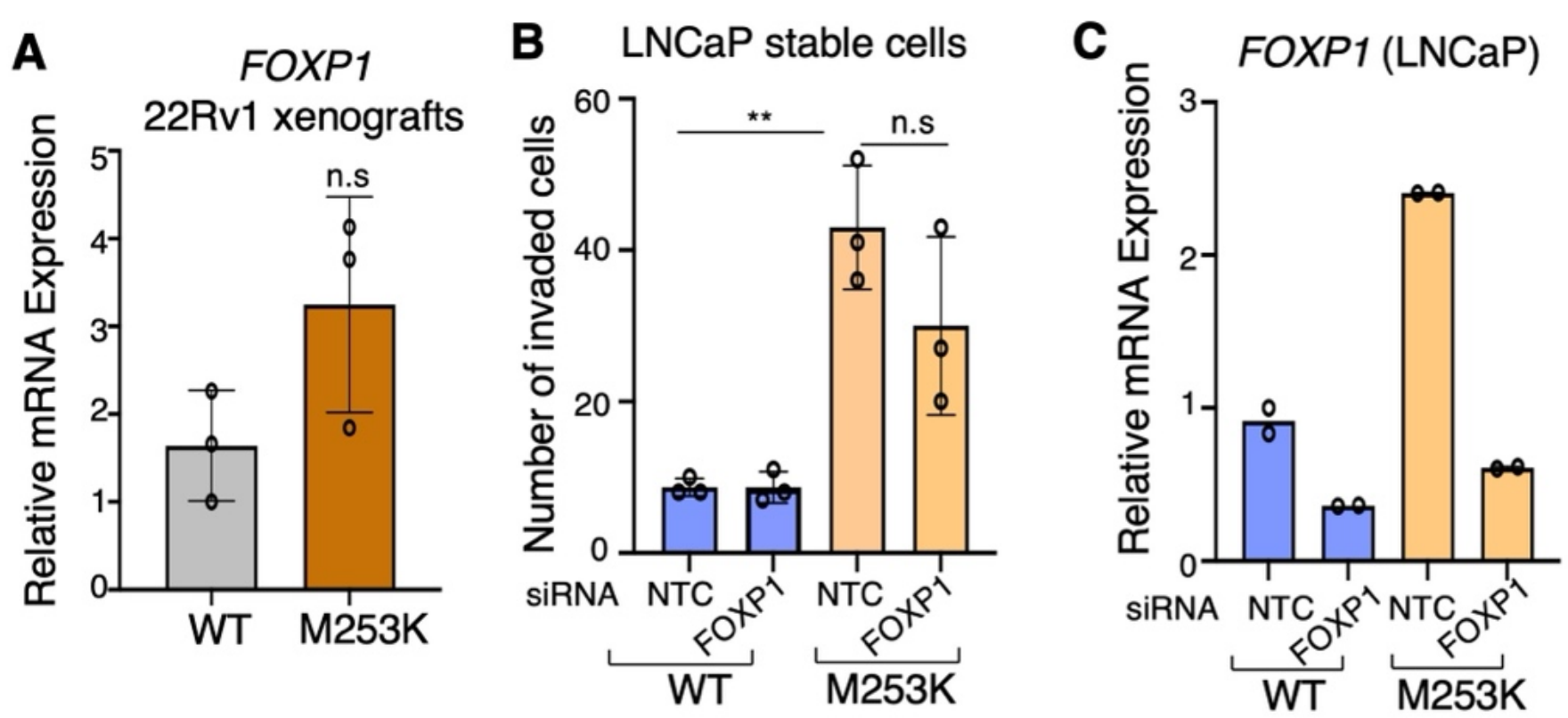

Supplementary Figure 3. Knockdown of FOXP1 effects on FKHD mutant cell invasion. A qRT-qPCR of FOXP1 in 22Rv1 mutant xenografts. **B**-**C** LNCaP stable cells were treated with siFOXP1. Invaded cells were counted (B), RT-qPCR of FOXP1 expression after knockdown (C).

A-B: n = 3 independent samples; data represented as mean ± SEM; statistical significance determined by unpaired two-sided t-test. C: n = 2 independent samples.

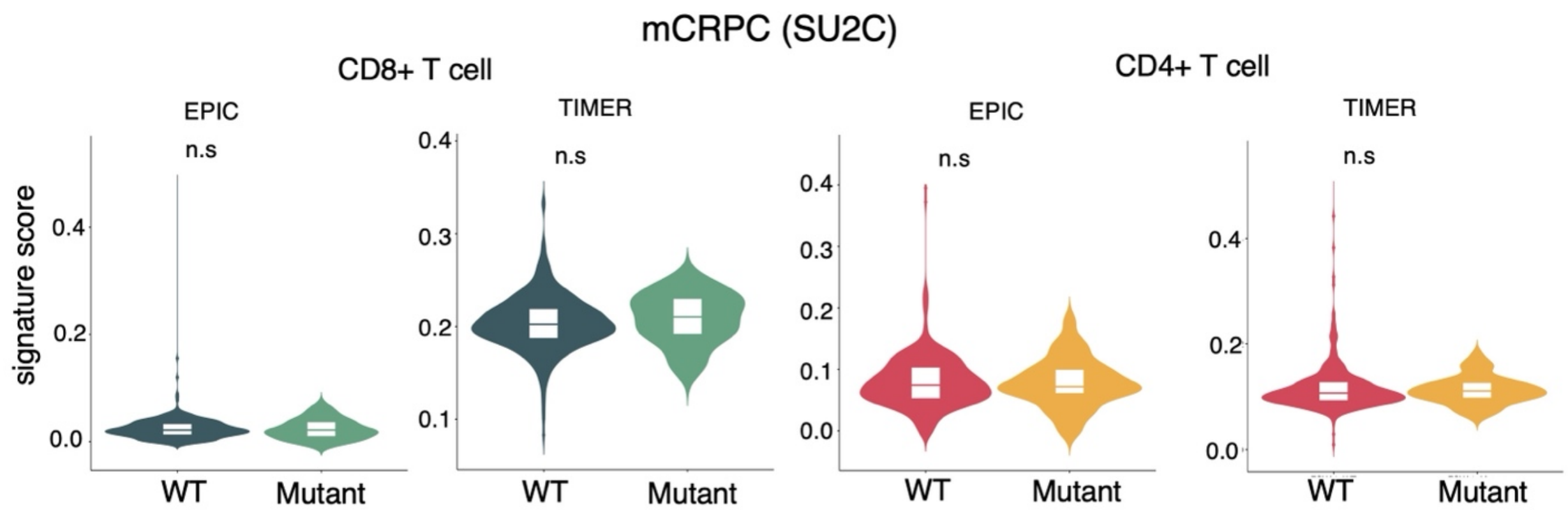

Supplementary Figure 4. Immune infiltrates in FOXA1 mutant CRPC patients.

Violin plot of immune infiltrates signatures in CRPC patients (SU2C cohort) (center: median; box: 25th to 75th IQR; shape: kernel density estimate, statistical significance determined by unpaired two-sided t-test).

Original blots related to Fig. 3H

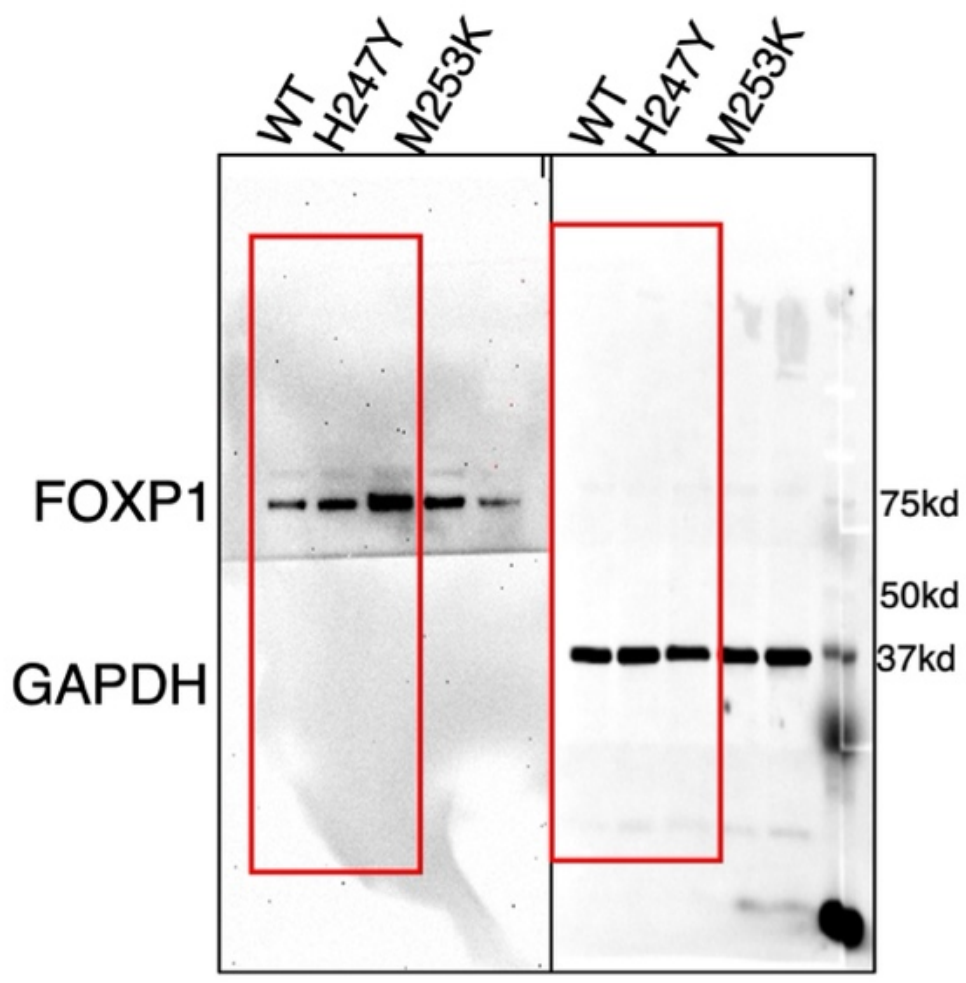

Original blots related to Fig. S1A

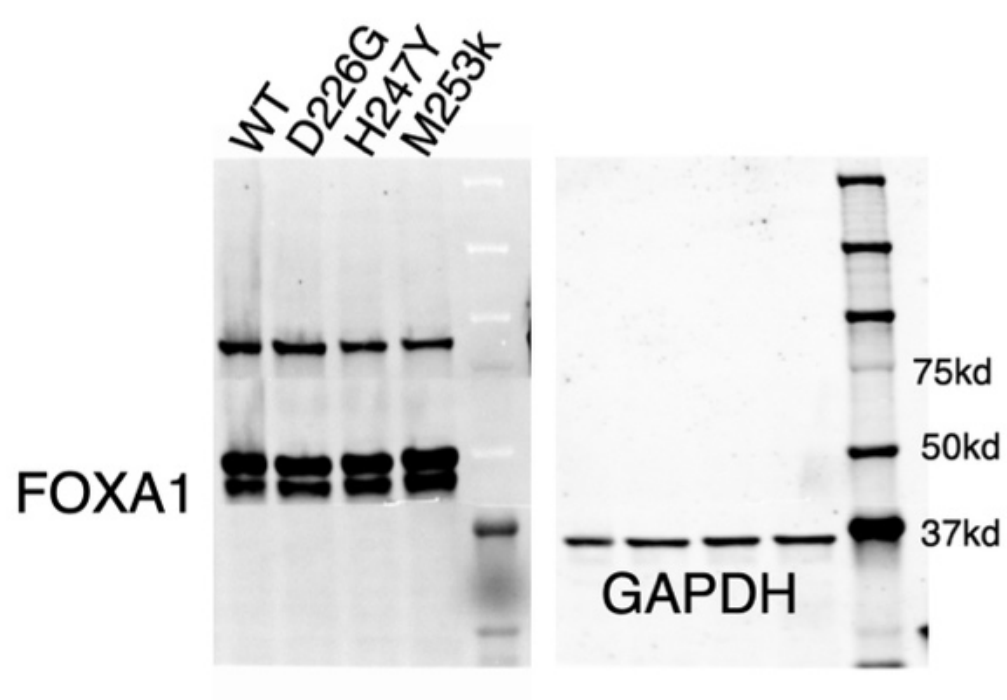

Original blots related to Fig. S1B

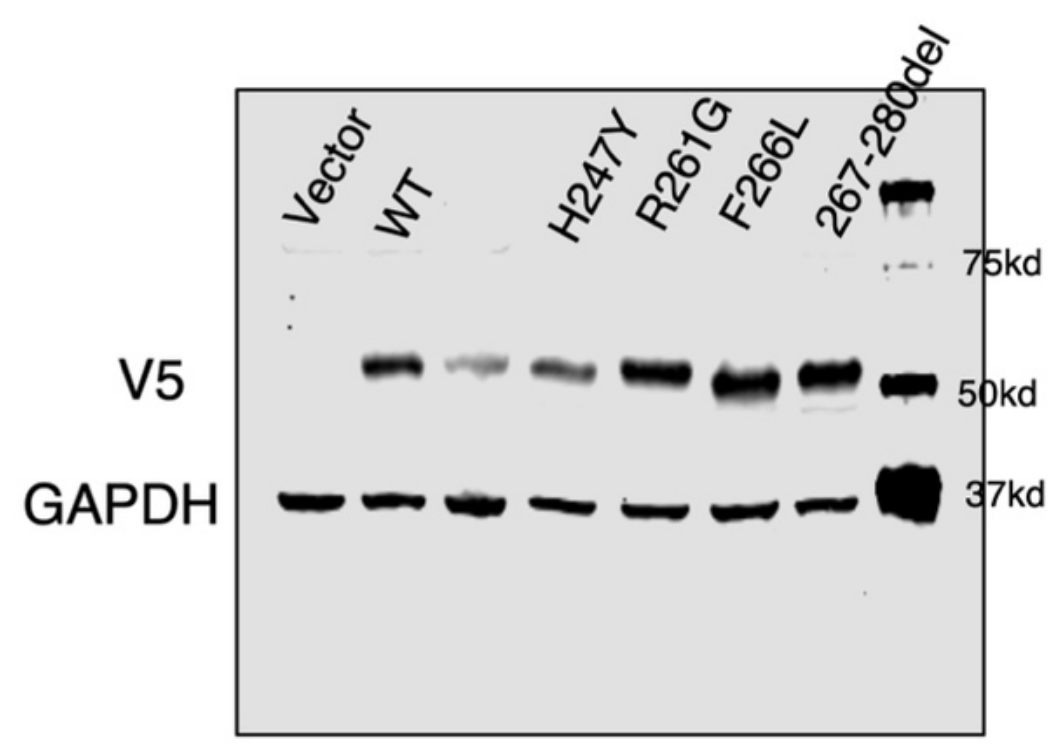

Supplementary Figure 5. Uncropped gel blots
