# Supplementary material for: Identification of targets of clinical FOXA1 mutants involved in EMT and immune suppression in prostate cancer
Source: Commun Biol. 2026 Jun 23;9:1326. doi: 10.1038/s42003-026-10523-2 (PMC13655023; doi:10.1038/s42003-026-10523-2)
Supplement: Supplementary file 2 [file 42003_2026_10523_MOESM2_ESM.pdf]

Description of Additional Supplementary File

File name: Supplementary Data
Description: raw data for the main figures.
